# Transcription Sites Are Developmentally Regulated during the Asexual Cycle of *Plasmodium falciparum*

**DOI:** 10.1371/journal.pone.0055539

**Published:** 2013-02-07

**Authors:** Carolina B. Moraes, Thierry Dorval, Mónica Contreras-Dominguez, Fernando de M. Dossin, Michael A. E. Hansen, Auguste Genovesio, Lucio H. Freitas-Junior

**Affiliations:** 1 Center for Neglected Diseases Drug Discovery (CND3), Institut Pasteur Korea, Gyeonggi-do, South Korea; 2 Cell Differentiation and Toxicity Group, Institut Pasteur Korea, Gyeonggi-do, South Korea; 3 Image Mining Group, Institut Pasteur Korea, Gyeonggi-do, South Korea; Tulane University, United States of America

## Abstract

Increasing evidence shows that the spatial organization of transcription is an important epigenetic factor in eukaryotic gene regulation. The malaria parasite *Plasmodium falciparum* shows a remarkably complex pattern of gene expression during the erythrocytic cycle, paradoxically contrasting with the relatively low number of putative transcription factors encoded by its genome. The spatial organization of nuclear subcompartments has been correlated with the regulation of virulence genes. Here, we investigate the nuclear architecture of transcription during the asexual cycle of malaria parasites. As in mammals, transcription is organized into discrete nucleoplasmic sites in *P. falciparum*, but in a strikingly lower number of foci. An automated analysis of 3D images shows that the number and intensity of transcription sites vary significantly between rings and trophozoites, although the nuclear volume remains constant. Transcription sites are spatially reorganized during the asexual cycle, with a higher proportion of foci located in the outermost nuclear region in rings, whereas in trophozoites, foci are evenly distributed throughout the nucleoplasm. As in higher eukaryotes, transcription sites are predominantly found in areas of low chromatin density. Immunofluorescence analysis shows that transcription sites form an exclusive nuclear compartment, different from the compartments defined by the silenced or active chromatin markers. In conclusion, these data suggest that transcription is spatially contained in discrete foci that are developmentally regulated during the asexual cycle of malaria parasites and located in areas of low chromatin density.

## Introduction

Almost half of the world population lives in areas of endemic malaria, a tropical disease that causes approximately 800,000 deaths every year. *Plasmodium falciparum*, the parasite that causes severe malaria, has a complex life cycle with several distinct stages of development, each characterized by unique morphological, physiological and molecular features.

Despite progress in sequencing the genome of the parasite [1] and studies of the transcriptome [2], [3], the molecular mechanisms of gene expression and regulation in malaria parasites remain poorly understood.

The extremely high AT content of the *P. falciparum* genome has frustrated the discovery of regulatory sequences by both bioinformatics methods and classical phylogeny, with a few exceptions [4]. As in many other eukaryotes, the *P. falciparum* genome encodes RNA polymerase II (RNA pol Il) basal transcription machinery and associated transcription factors (TAFs – [5]), whereas orthologs of other canonical transcription factors have not been found. However, the apparent absence of transcription factors in *Plasmodium* has been challenged by the identification of the first family of putative transcription factors specific to Apicomplexa, the ApiAP2 [1], [6], [7].

Epigenetic factors, such as histone post-translational modifications, gene positioning and nuclear organization, have also been consistently shown to play a role in controlling gene expression in *P. falciparum*. The first direct evidence for the involvement of spatial organization of the genome in gene regulation was the discovery that *var* genes are silenced when associated with telomeric clusters [2], [3], [8]–[11]. The nuclear periphery was also identified as a zone of silencing, formed by an electrondense structure that suggests the presence of heterochromatin [1], [9], and is associated with the silencing factor PfSir2A and the heterochromatin marker PfHP1 [4], [11], [12]. The unique location of these proteins in perinuclear zone occurs along with the transcription of *var* genes in this region, suggesting that there is at least one area in the periphery where genes can be activated amid repressive domains. It is a point of contention whether *var* gene activation can occur in association with the telomeric cluster [5], [10], [13], or whether it requires the translocation of the *var* gene to a transcriptionally active area in the nuclear periphery [9], [14].

Among the nuclear compartments defined by the presence of specific proteins, the nucleolus is the best characterized currently. *P. falciparum* has a single peripheral nucleolus defined by the protein PfNop1, also known as fibrillarin [15]. PfSir2A, PfTERT, which encodes the protein component of telomerase, and PfOrc1 are associated with the nucleolus [11], [15], [16]. Other nuclear proteins, as well as specific histone modifications, all of which are associated with the activation/repression of transcription, are located in discrete nuclear compartments [12], [17], [18]. The fact that most of these compartments are topographically distinct demonstrates that the nucleus of *P. falciparum* is highly structured spatially. However, a functional characterization of these sub-nuclear compartments is needed to better understand the relationship between nuclear architecture and nuclear processes such as transcription.

Most studies on the spatial organization of transcription were performed in mammalian cells. In permeabilized HeLa cells, the incorporation of 5-bromouridine 5′-triphosphate (BrUTP) demonstrated for the first time that transcription is organized into discrete foci throughout the nucleus [19]–[21]. Subsequent studies demonstrated that these foci contain various transcription units [22], [23]. These foci were dubbed “transcription factories” because they are aggregates of genes, polymerases and transcription factors, and it has been argued that this proximity greatly increases the efficiency of transcription. It has further been proposed that transcription factory structures are conserved throughout evolution and provide the “molecular ties” involved in the maintenance of nuclear genome architecture (reviewed by [24]).

Given the importance of spatial organization of chromatin and nuclear compartmentalization for malaria parasite gene regulation, we sought to quantitatively and qualitatively characterize the spatial organization of global transcription in *P. falciparum*. Here, we report that transcription is organized in foci developmentally regulated in terms of both number and location during the asexual cycle. Transcription mostly occurs in areas of low chromatin density, in a novel compartment, distinct from some of the subcompartments previously described in the *P. falciparum* nucleus.

## Materials and Methods

### Culture and Synchronization

The *P. falciparum* 3D7 strain was maintained in RPMI 1640 medium supplemented with sodium bicarbonate (Welgene #LM011-05) and 3% hematocrit (type O human erythrocytes, Rh+) cultured at 37°C in an atmosphere of 1% O_2_, 5% CO_2_ and 94% N_2_. For routine maintenance of cultures, parasitemia was kept between 0.5 and 5%. Prior to BrUTP incorporation experiments (described below), parasites were subjected to gelatin purification of mature stages (adapted from [25]) followed by sorbitol synchronization [26]. Briefly, unsynchronized cultures were centrifuged at 1000×*g* for 2 min at room temperature, and each pellet was resuspended in 9 volumes of 0.7% gelatin (Sigma, #G2625) in RPMI and incubated at 37°C for 1 h. The supernatant containing only mature parasites (ideally mostly schizonts at approximately 36–40 hours post-invasion (hpi)) was collected in a fresh tube, washed once in RPMI, and placed in culture. Fresh red blood cells were added to a final hematocrit of 3%, as described above. The cultures were gassed and incubated at 37°C for 4 hours to allow the invasion to proceed (the erythrocytic cycle of the 3D7 strain lasts approximately 40 to 44 hours), and after this period, the culture was treated with 5% sorbitol, in a proportion of 4 volumes of 5% sorbitol to 1 volume of pelleted red blood cells (RBCs), for 5 min at 37°C. This treatment is intended to lyse mature forms, which did not complete their developmental cycle. Young stages (in this instance, ring forms at 0–4 hpi) are not affected by this treatment. The pellet containing only ring-infected and non-infected RBCs was washed in culture media and cultured for an additional period as needed. For the BrUTP labeling experiments, cultures were used at 4 points in their erythrocytic cycle: early rings from 0 to 4 hpi (for simplicity, referred to as 2 hpi, which is the average developmental age of the population), rings from 8 to 12 hpi (referred to as at 10 hpi), trophozoites from 20 to 24 hpi (referred to as at 22 hpi) and schizonts from 32 to 36 hpi (referred to as 34 hpi). Typically, two rounds of gelatin/sorbitol synchronization (treatments in two consecutive erythrocytic cycles) are needed to achieve a high degree of synchronization in the population (approximately more than 90% of parasites at the same phase of the cycle).

### *In situ* Nascent RNA Labeling

The incorporation of 5-bromouridine 5′-triphosphate (BrUTP) in *P. falciparum* was adapted from a previously published report [27]. The synchronized culture was collected at 10 and 22 hpi, centrifuged at 1000×*g* for 2 min at room temperature, and resuspended at 1×10^9^ infected red blood cells (iRBC)/ml in ice-cold wash buffer (150 mM sucrose, 20 mM potassium glutamate, 3 mM MgCl_2_, 2 mM DTT and 10 µg/ml leupeptin). Lysolecithin (Sigma, #L4129) was added to a final concentration of 0.01%, and after homogenization, cells and parasites were permeabilized for 5 min in an ice water bath, then centrifuged at 2500×*g* for 10 min at 4°C and washed 3 times in cold wash buffer. Cells were resuspended at 1×10^9^ iRBCs/ml in cold wash buffer either with or without the addition of 200 µg/ml of α-amanitin and incubated for 5 min at 37°C. Subsequently, 2 mM ATP, 1 mM GTP, 1 mM CTP and/or 1 mM BrUTP (final concentrations) were added to both preparations, and nucleotide incorporation was allowed to proceed for 10 min at 37°C. The parasites were then immediately fixed in 4% paraformaldehyde in PBS at pH 7.4 (4% PFA) and at a density of 1.25×10^8^ iRBCs/ml for 20 min at room temperature. After fixation, the parasites were centrifuged at 2500×*g* for 10 min at 4°C, washed once in PBS and placed onto a microscope slide in a humid chamber at room temperature for 30 min to allow parasites to adhere to the glass. Fluorescent microspheres (Tetraspeck, Invitrogen, #T7283) were included in the sample preparation to be used as reference for scale and Z image alignment during automated image analysis (see below), in case of confocal Z series.

### Immunofluorescence Assay

Parasites on glass slides (prepared as described above) were permeabilized in 0.1% Triton X-100 in PBS for 2 min at room temperature, washed 3 times in PBS and blocked for 30 min in 4% BSA in PBS pH 7.4 (4% BSA), followed by primary antibody incubation. An anti-deoxyuridine (anti-BrdU) mouse monoclonal antibody (clone PRB1, Invitrogen, cat. no. A21300) was used for the detection of BrUTP-labeled nascent RNA (BrRNA); rabbit polyclonal serum was used for the detection of PfSir2A [11], (kindly provided by Dr. A. Scherf, Institut Pasteur, France); goat anti-human fibrillarin serum (Santa Cruz Biotechnology, cat. no. SC-11335) was used for detection of PfNop1; and rabbit polyclonal serum was used to detect modified histones, acetylated histone H4 (anti-acetyl histone H4 antibody or anti-H4ac, Millipore, cat. no. 06-598) and histone H3 trimethylated at lysine 79 (anti-Histone H3 trimethyl-K79 antibody or anti-H3K79me3, Abcam, cat. no. AB2621). All primary antibodies were diluted 1∶50 (v/v) in 4% BSA, with the exception of anti-acetyl histone H4 antibody, which was diluted 1∶100 (v/v). After incubation with primary antibodies, parasites were washed 3 times in PBS and incubated with an appropriate labeled secondary antibody and 5 µg/ml DAPI diluted in 4% BSA for 30 min. Alexa Fluor 488 goat anti-mouse IgG (Invitrogen, cat. no. A11001), diluted 1∶200 (v/v), was used to detect the anti-BrdU antibody alone or in combination with anti-PfSir2A, anti-H4ac and anti-H3K79me3; Alexa Fluor 555 goat anti-rabbit IgG (Invitrogen, cat. no. A21429), diluted 1∶800 (v/v), was used to detect anti-PfSir2A, anti-H4ac and H3K79me3; and Alexa Fluor 555 donkey anti-mouse IgG (Invitrogen, cat. no. 31570) and Alexa Fluor 488 donkey anti-goat IgG (Invitrogen, cat. no. A11055), both at 1∶200 dilution (v/v), were used for the simultaneous detection of mouse anti-BrdU and goat anti-human fibrillarin. After incubation with secondary antibody and DAPI, the parasites were washed three times in PBS, and the samples were mounted in Vectashield (Vector Labs, cat. no. H-100) and sealed.

### Image Acquisition

Two-dimensional images were obtained using a Nikon Eclipse 90i microscope equipped with an oil immersion 100×/1.4 PlanApoVC objective and the quantitative Nikon DS-QiMc camera controlled by NIS-Elements AR software. Images were acquired in gray scale in the quantitative range of intensity. Three-dimensional images (Z series) were acquired on a confocal microscope equipped with a 63×/HCX 1.4 PL Apo lbdBL oil immersion objective. The diaphragm was set to 1.00 *airy*, and both channels were acquired simultaneously (for imaging of DAPI combined with Alexa Fluor 555 staining) or sequentially (for imaging of DAPI combined with Alexa Fluor 488 staining) using two independent photon multipliers (PMTs). A 405 nm diode laser, a 488 nm argon laser, and a 561 nm DPSS green laser were used for the excitation of DAPI, Alexa Fluor 488 and 555, respectively, and emission was acquired at 415–485 nm for DAPI: 515–570 nm for Alexa Fluor 488 and 570–645 nm for Alexa Fluor 555. Each Z optical slice was composed of an average of 16 frames at 512×512 pixels, 8 bits, with the size of the voxel (a three dimensional pixel) corresponding to 58×58×122 nm. After acquisition, 2D and 3D images were assembled using the ImageJ software [28]. Images were treated for the removal of background using the “rolling ball” function of ImageJ with the radius set at 50 pixels.

### Image Segmentation

A significant level of precision is required for detection and localization of sites of RNA synthesis inside the nucleus of the parasite. To increase the relevance of image processing, images were acquired with a signal-to-noise ratio (SNR) as high as possible, mainly due to the low level of fluorescence of microscopic subnuclear structures of *P. falciparum*. The SNR was optimized by increasing the number of images acquired and averaging them to 16 frames per Z optical slice. To ensure that at the end of long acquisition periods the Z slices were correctly assembled in 3D, fluorescent microspheres were used as references by a customized algorithm that removes intrinsic microscope stage motion and chromatic aberrations, which are negligible in most applications but became important in the case of imaging the miniscule *P. falciparum* nucleus [29]. After corrected 3D images were assembled, the nuclei were segmented based on a semi-automatic process (modified from [30]). A user-selected region of interest (ROI), which contains a single nucleus, was submitted to automatic thresholding by the K-means clustering algorithm. Voxel classification into the fore- or background was performed based on the nearest neighbors method, followed by nuclei segmentation using a virtual 3D mesh filtered by a Gaussian smoothing kernel. This step gave greater certainty to the nuclear segmentation and enabled a confident estimation of the nuclear center and volume. Transcription foci were identified by the maximum local curvature after convolution with a Gaussian kernel, determined empirically to be 2 µm. Using the finite difference method, a map of the Gaussian curvature was then computed in 3D, and the local maxima curvatures were defined as sites of transcription. The transcription foci curvature map was overlaid on the nuclear mesh, allowing for the determination of the nuclear radial position of each transcription site. The intensity of the sites were measured and normalized based on the PMTs gain used during acquisition.

### Nuclear Positioning of Transcription Foci

To quantitatively compare transcription foci positions across different nuclei and different experiments, each focus radial position was normalized to its respective nucleus radius. Briefly, the nucleus ellipsoid was projected onto a sphere of radius = 1, and the positions of the sites were determined and relativized in relation to this radius. Then, three concentric nuclear zones of equal volume were defined.

### Statistical Analysis

Unpaired *t*-tests were performed using the software GraphPad Prism version 4.03 (www.graphpad.com). *p* values less than 0.05 were considered significant. For the analysis of transcription sites radial distribution, we applied the method described by [31].

## Results

### The Automated Analysis of 3D Images Indicated that Transcription Sites are Dynamic Structures Reorganized during the Asexual Cycle

**Figure 1:**
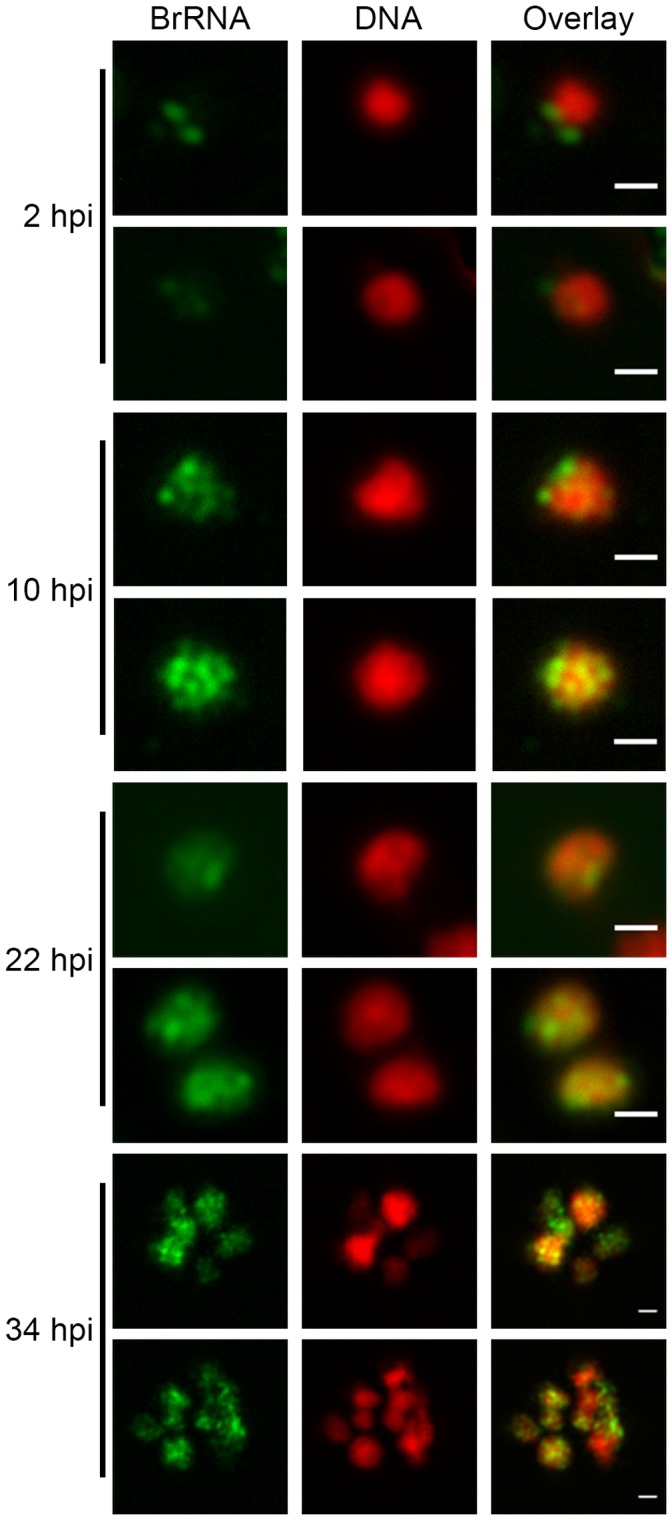
Transcription occurs in discrete foci in the nucleus of asexual forms of malaria parasites. Synchronized populations of *P. falciparum* were labeled with BrUTP, and nascent RNA (BrRNA, in green) was detected by indirect immunofluorescence, showing that transcription sites are organized in foci distributed throughout the nucleus in early rings (2 hpi), rings (10 hpi), throphozoites (22 hpi) and schizonts (34 hpi). DNA was stained with DAPI and artificially colored in red for enhanced contrast. Representative images are shown. Bars, 1 µm.

Transcription can be visualized *in situ* by the incorporation of modified nucleotides, such as 5-bromouridine 5′-triphosphate (BrUTP) in permeabilized cells. Given our interest in studying the spatial organization of transcription in *P. falciparum*, we standardized the technique of incorporation of BrUTP for this parasite. This technique was difficult to perform because, among other factors, the parasite is extremely sensitive to permeabilization. It is also noteworthy that we attempted to standardize the incorporation of bromouridine (BrU) in cultures (i.e., intact iRBCs), but *P. falciparum* in culture did not incorporate BrU in nascent RNA, at least at levels detectable by immunofluorescence (data not shown). As shown in Figure 1, BrUTP can be specifically incorporated into the nascent RNA of asexual forms of *P. falciparum*. In the earliest stages of the asexual cycle (2 hpi), nascent transcription is visualized mostly as a few, low-intensity spots in the periphery of the nucleus; as the parasite progresses in the asexual cycle, in rings at 10 hpi and trophozoites at 22 hpi, a higher number of transcription foci is seen. It is also possible to visualize transcription foci in several nuclei of segmented schizonts, thus demonstrating that this technique enables the sensitive and specific detection of transcription throughout the asexual cycle.

The incorporation and detection of BrUTP into nascent RNA (BrRNA) can be inhibited by the treatment of permeabilized cells with the RNA polymerase II inhibitor α-amanitin, leaving only the RNA polymerase I transcription foci to be visualized (Figure S1). As expected, one to two foci can be seen in each nucleus; these are the sites of asexual rRNA transcription ([32]). Incubation of permeabilized cells with BrUTP without ATP, GTP or CTP also demonstrates that this technique detects specifically nascent BrRNA and not BrUTP unspecifically bound to nuclear structures (Figure S1).

The majority of *P. falciparum* genes expressed during the asexual cycle are developmentally regulated [2], [3]. The data shown in Figure 1 suggest that different asexual cycle stages appeared to have different numbers of nascent RNA foci. These findings, which are in accordance with a previously published report [32], suggest that transcription sites could also be developmentally regulated during the asexual cycle.

A customized image analysis algorithm was developed to quantitatively characterize the number and distribution of transcription sites in the nuclei of different asexual forms of *P. falciparum.* The use of automated analysis of subnuclear structures enables the objective and unbiased detection of the object studied [30], [33]. The developed software allows for the quantification of transcription sites from the identification of local peaks of intensity (curvature) in the BrRNA signal in 3D confocal series. Furthermore, the software is able to determine the radial position and intensity of each site in a given nucleus, as described in the Materials and Methods section.

**Figure 2:**
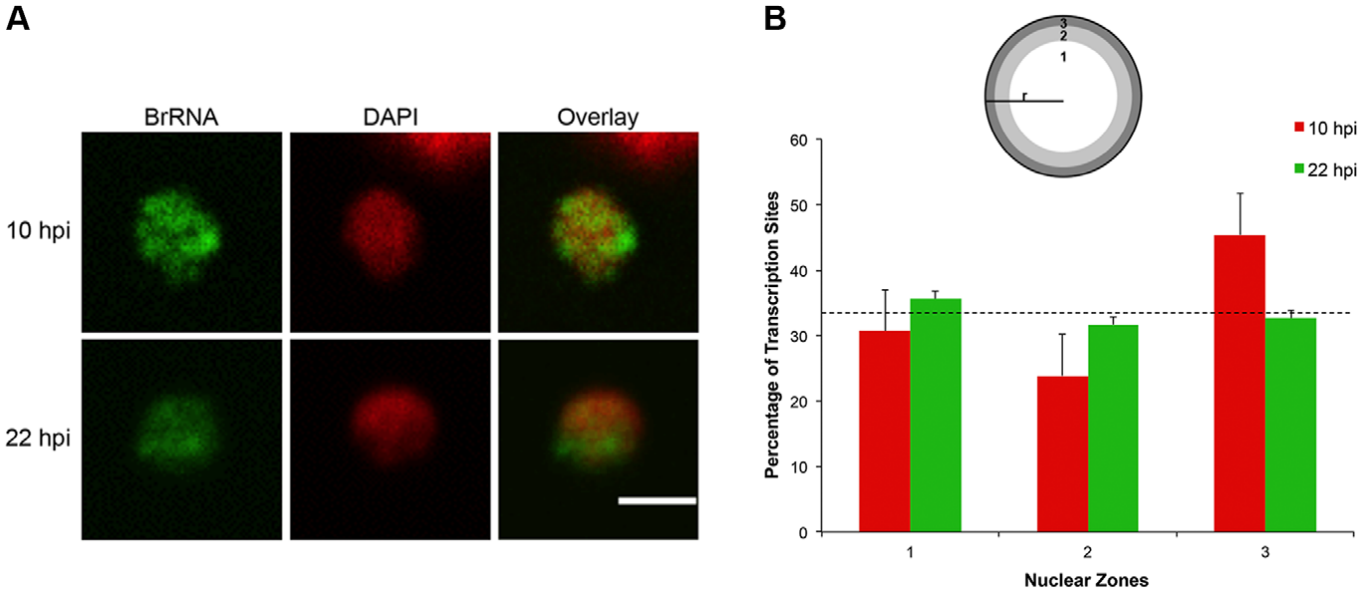
RNA synthesis is spatially remodeled during the asexual cycle. (A) Representative confocal images of rings (10 hpi, top) and trophozoites (22 hpi, bottom) used for automated image analysis of transcription site number and distribution in the asexual cycle. Images are maximal-intensity 2D projections of confocal Z-series. Bar, 1 µm. (B) Images of nuclei from confocal Z-series of rings (10 hpi) and trophozoites (22 hpi) were automatically segmented in 3D and project onto spheres of normalized radius “r” equal to 1 (scheme, top). The nucleus was divided into 3 concentric zones of equal volume, where zone 1 was the most internal zone (projected in 2D and represented in white in the circle), followed by the intermediate zone 2 (light gray) and the outermost zone 3 (dark grey); the probability of a transcription site randomly being in one of the three zones is 33.3%. Transcription sites were detected by automated image analysis, and individual site positions denote the normalized nuclear radial position. The graph shows the distribution of sites per nuclear zone in the 10 hpi ring (red) and 22 hpi trophozoite (green) populations, showing that while in rings transcription occurs mostly at the peripheric zone (p = 3.34×10^−6^), it is equally distributed in the three nuclear zones in trophozoites (p = 0.3324).

Using synchronized populations, we acquired confocal Z series of parasites labeled by BrUTP in two distinct stages of the erythrocytic cycle: ring (10 hpi) and trophozoite (22 hpi – Figure 2A), and the series were submitted to automated image analysis. The low intensity of transcription foci in early rings (2 hpi) and the complex curvature of schizont nuclei hampered the automated analysis of transcription foci at these asexual stages (data not shown).

The appearance and average number of transcription sites were found to be markedly different in the two phases: at 10 hpi, transcription sites appeared more as individualized foci, while at 22 hpi, sites appeared more juxtaposed and less individualized, with the BrRNA signal radiating from the center of the sites to the nucleoplasm (Figure 2A). The number of transcription foci per nucleus was also different: at 10 hpi, the parasites presented in average 9 detectable foci per nucleus, while at 22 hpi, there were on average 6 foci per nucleus. This difference was statistically significant (*p*<0.0001; see Table 1).

**Table 1:** Quantification of transcription sites and nuclear volume in the asexual cycle.

Stage	*n* nuclei	*n* sites	Ave. no. sites per nucleus (SD)	Ave. nuclear volume, µm^3^ (SD)	Ave. site intensity, A.U. (SD)
10 hpi	58	502	9 (4)	5.3 (1.7)	1.7 (1.0)
22 hpi	108	621	6 (2)	5.2 (1.6)	3.9 (2.1)

Ave., average; SD, standard deviation; A. U., arbitrary units.

It has been demonstrated that for higher eukaryotes, the number of transcription sites in one nucleus is proportional to the nuclear volume [23]. Therefore, we asked whether the changes in the number of transcription sites in *P. falciparum* detected at 10 and 22 hpi would correlate with changes in nuclear volume to maintain a constant density of transcription sites per unit of nuclear volume. However, the average nuclear volume between 10 and 22 hpi remains virtually unchanged (approximately 5 µm^3^), and the difference in the average volume between the two populations is not statistically significant (p = 0.807– Table 1). Therefore, this analysis suggests that in *P. falciparum*, the nuclear density of transcription sites does not remain constant during the asexual cycle. Furthermore, changes in the number of transcription sites can occur independently of changes in nuclear volume, suggesting that other factors might drive the assembly of transcription sites. The data also suggest that such factors would also be developmentally regulated.

It has been proposed that the nuclear periphery of *P. falciparum* is primarily a silencing area as it contains a heterochromatin-like structure and is associated with the silencing factor PfSir2A and the heterochromatin markers PfHP1 and H3K9me3 [9], [11], [12], [14]. However, we noticed that several transcription foci seemed to be located in the nuclear periphery. To determine the spatial distribution of transcription sites and evaluate whether they were randomly scattered in the nuclear space, the distance of each transcription site center to the nuclear center was measured, normalized to the nuclear radius, and scored for one of the three concentric nuclear regions of equal volume. If transcription sites were randomly distributed, there would be an equal chance of finding a given transcription focus in any of the concentric zones.

Unexpectedly, analysis of the nuclear distribution demonstrated that there is spatial reorganization of transcription sites during the erythrocytic cycle (Figure 2B): whereas sites are distributed throughout the nucleus in both rings and trophozoites, at 10 hpi, more sites (approximately 45% of all sites) were located in the outer nuclear region. This distribution pattern was not random (*p = *3×10^−6^), contrasting with the distribution of sites at 22 hpi, when they were almost evenly dispersed among the three nuclear concentric zones of equal volume (*p = *0.332). Altogether, the data suggest that transcription sites are dynamic structures that are spatially and developmentally regulated during the asexual cycle of *P. falciparum*.

### Transcription Occurs Preferentially in Areas of Low Chromatin Density

**Figure 3:**
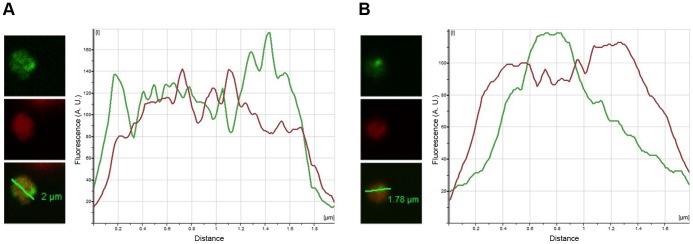
Transcription sites localize preferentially in zones of lower chromatin density. A single confocal Z slice picture was analyzed for the intensity profile for both BrRNA (green, top images) and DNA (red, middle images) along the line drawn in green over the 2-channel composite picture (Overlay, bottom images). The length of the green line is indicated. The graphs show the fluorescence intensity profile of transcription sites and DAPI along the line for 10 hpi rings (A) and 22 hpi trophozoites (B). Note that peaks of intensity for BrRNA occur in regions where there is a decrease in intensity for the DAPI signal, and vice-versa, suggesting that the preferred sites for transcription occur in the areas of lower chromatin intensity. A. U., arbitrary units.

We also observed that the most intense transcription sites were often located in areas of low DAPI labeling. Analysis of the relationship between the BrRNA and DAPI signals reveals that these two markers are inversely related and that BrRNA signal peaks, which correspond to transcription sites, are located in areas of low DAPI signals, for both 10 hpi (Figure 3A) and 22 ηπι (Figure 3B). These results suggest that transcription occurs preferentially in a nuclear compartment characterized by low chromatin density. In mammals, it is has been shown that the outer layers of a transcription site are in contact with the chromatin, whereas the central region of the sites is occupied only by BrRNA and proteins, but not DNA [34]–[36]. In this context, our data indicate that regions of low intensity of DAPI in *P. falciparum* correspond to regions of low chromatin density. The data also suggest that although the number and distribution of transcription sites in the malaria parasite nucleus differ from those found in higher eukaryotes, the compartmentalization of transcription in discrete sites that differ from chromosomal regions is conserved in *P. falciparum.*

### Transcription Sites Define a Distinct Subnuclear Compartment

**Figure 4:**
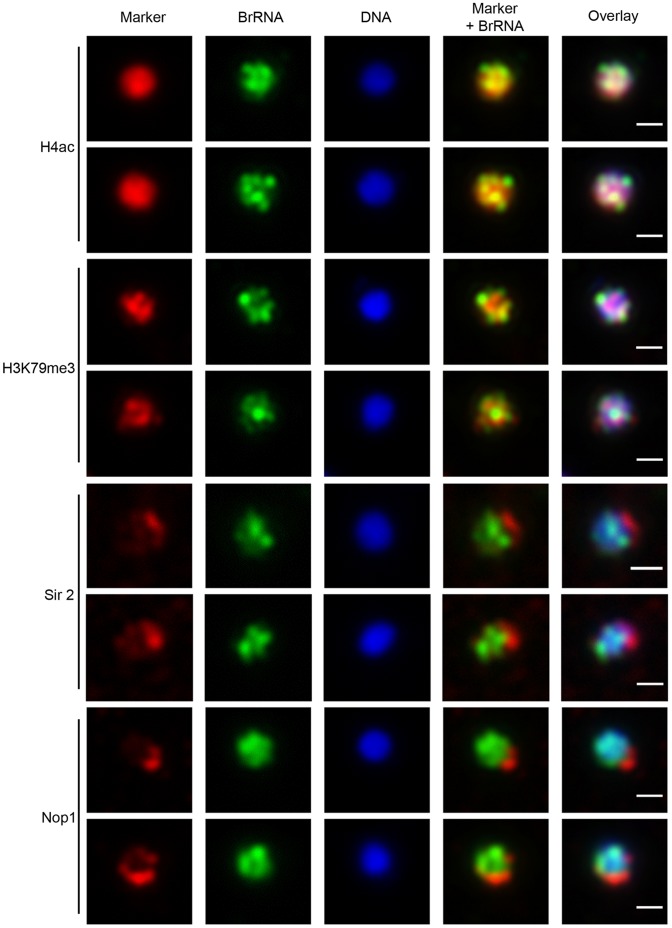
Transcription sites form a distinct compartment in the nucleus of *P. falciparum*. Immunofluorescence assay for nascent RNAs of ring stage parasites labeled with BrUTP (BrRNA, green) and the compartments of acetylated H4 (H4ac, active chromatin); a proposed histone marker of nascent transcription (H3K79me3; [17]); PfSir2A (silencing compartment); and PfNop1 (nucleolar compartment). DNA was labeled by DAPI, in blue. Bars, 1 µm.

The nucleus of *P. falciparum* is highly subcompartmentalized. Given this scenario, we probed the relationship between the transcription sites and some of the characterized subcompartments of *P. falciparum*. We analyzed the distribution of the nucleolar and telomeric cluster marker PfNop1 [15]; the active chromatin marker acetylated histone H4 (H4ac [11]); and the marker for histone H3 trimethylated at lysine lysine 79 (H3K79me3), a chromatin modification, which has been proposed as a potential transcription site marker [17]. Additionally, due to the peripheral distribution of some transcription sites, we examined the spatial relationship between transcription and the silencing factor PfSir2A, a histone deacetylase, which is distributed at the nuclear periphery and in the nucleolus and is capable of silencing virulence genes such as *var* and *rif* [8], [11], [37]. Interestingly, as seen in representative examples in Figure 4, although H4ac stains almost the entire nucleus and therefore spans across transcription sites as well, the labeling pattern of both markers is consistently different, demonstrating that the general acetylation of histone H4 is not indicative of ongoing transcription in *P. falciparum.* We also consistently observed one transcription site that is peripheric and seemed to be excluded from the H4ac domain, further reinforcing the concept that general acetylation is not a marker of ongoing transcription. Likewise, the marker H3K79me3 showed little to no colocalization with transcription sites, suggesting that this specific histone H3 modification is not associated with active transcription.

The silencing factor PfSir2A is found in foci at the nuclear periphery, telomeric clusters and nucleolus [11]. Because we observed transcription sites at the nuclear periphery, we decided to investigate the relationship between transcription sites and silent chromatin as defined by the presence of PfSir2A and between transcription sites and the nucleolus and telomeric clusters as defined by the presence of PfNop1.

The compartment defined by PfSir2A appears to be formed by two distinct components, which showed an interesting pattern in relation to transcription sites: one PfSir2A area, which was strongly labeled and clearly localized to one pole of the nuclear periphery did not colocalize with transcription sites, which are located on the opposite pole of the nucleus; the other areas, which were faintly labeled and formed foci suggestive of telomeric clusters, exhibited a partial overlap with transcription sites (Figure 4). The nucleolar/telomeric cluster marker PfNop1 had a labeling pattern similar to that of PfSir2A, and in this instance, there was only a partial overlap between the transcription foci and the PfNop1 compartment. These results bolster the view that the nucleus is highly compartmentalized and accommodates antagonistic functions such as transcription and silencing in the same or juxtaposed areas. Regardless, however, none of the analyzed chromatin markers strictly reflects the active transcription compartment, and further experiments are needed to investigate if any of the several nuclear markers previously described for *P. falciparum* correspond to a marker of ongoing transcription.

## Discussion

Gene expression in *Plasmodium falciparum* is the product of several layers of control [38]. Among these, nuclear architecture has increasingly been pointed to as an epigenetic factor crucial in *P. falciparum* gene regulation. In this work, we quantitatively studied the overall spatial organization of nascent transcription in malaria parasites. Using a customized computer algorithm, the number and distribution of transcription sites in the nucleus of *P. falciparum* were analyzed. This automated image analysis method has the advantage of allowing for unbiased transcription site determination and counting. Surprisingly, the average number of transcription sites, as well as their nuclear distribution, change during the asexual cycle, suggesting that the transcription compartment is a dynamic, developmentally regulated structure. Rings at 10 hpi have a higher number of transcription sites than trophozoites at 22 hpi, and this change in site number does not correlate with changes in nuclear volume between the two stages. The higher number of sites found in rings compared to trophozoites contrasts with the higher measured intensity of transcription sites in trophozoites than in rings, suggesting that transcription sites in trophozoites have more mRNA than those in rings. This interpretation is in agreement with the fact that trophozoites have a higher amount of mRNA than rings [39]. These results suggest that transcription sites are assembled upon the need for transcription, in accordance with the view that transcription is highly periodic and developmentally regulated during the asexual cycle.

Transcription of *var* genes occurs at the periphery of the nucleus [8], [9], [13], [14], [40], suggesting that, despite the presence of a heterochromatin-like structure and silencing factors, there is some transcriptional permissiveness in this region. Here, we show that the nuclear periphery may be the preferred region for transcription of genes other than *var* in the ring stage. The periphery is not, however, the region of preferential transcription during the whole asexual cycle, and in trophozoites, the central, intermediate and peripheral regions have the same probability of containing transcription sites. However, as the experimental procedures for BrUTP labeling of nascent transcripts require cell permeabilization, we cannot rule out unknown effects of permeabilization on the observed spatial distribution of transcriptional foci.

The fact that there are far fewer transcription sites than the estimated number of genes transcribed in each stage [2] suggests that (i) different genes must share sites and (ii) the number of transcription sites is limited and at least one order of magnitude fewer than the number of transcribed genes in a given stage. An elegant work by Deitsch and colleagues demonstrated that an increase in the copy number of the *var* promoter leads to downregulation of endogenous *rif, stevor* and *Pfmc-2TM*, implying that these gene families share the same regulators/activators/transcription factors and that these are limited in number [40].

Based on the hypothesis that genes must share a transcription site to be transcribed, several possibilities can be entertained including whether gene clustering at transcription sites are stochastic or deterministic events and whether this potential organization is linked to the periodic regulation of genes during the asexual cycle. In this sense, it would be of fundamental importance to evaluate how transcription sites relate to the recently described putative plant-like transcription regulators apetala 2 (ApiAP2) [6], [7]. Additionally, experiments of fluorescent in situ hybridization (FISH) and of chromatin conformation capture would be crucial in determining how co-activated genes are positioned relative to both one other and transcription sites. However, these experiments are technically challenging and difficult to perform.

We also observed a redistribution of transcription sites in different nuclear areas from rings to trophozoites: in rings, transcription occurs mostly in the outermost third of the nucleus, whereas in trophozoites, transcription sites are also distributed in the central, intermediate and peripheral nuclear areas. This is also the first report to our knowledge to quantitatively demonstrate that transcription sites may have a preferential localization in a nuclear zone, as mammalian transcription sites are distributed throughout the nucleus [23]. In this same study, the authors also reported that there is no change in the random pattern of distribution of the nuclear sites during the differentiation of stem cells from mice in parietal endoderm. Therefore, the fact that transcription sites in *P. falciparum* undergo spatial redistribution between 10 and 22 hpi is quite surprising and reinforces the notion that transcription sites are plastic structures, which are regulated differently during the process of development/differentiation that occurs in the asexual cycle.

We observed that transcription occurs in nuclear areas with a low DAPI labeling and assumed that these areas were poor in chromatin. The same is true for mammalian cells, in which transcription has been shown to be located at the surface of chromosomes, in the so-called interchromatin space [41]. Electron microscopy experiments would provide further evidence on how transcription sites relate to the chromatin and the interchromatin space, a nuclear compartment that has not yet been described in *P. falciparum.*

Our data also demonstrate that transcription in *P. falciparum* spatially defines a nuclear compartment different from the compartment defined by PfSir2A, PfNop1 and active chromatin histone mark H4ac. Apparently, H4ac is distributed throughout the nucleus and appears to mark all chromatin visualized by DAPI, suggesting that the state of chromatin acetylation is insufficient to determine the transcription of a gene in *P. falciparum*. These observations are in accordance with what has been described in [42]. In this study, the authors demonstrated that the promoters of genes transcribed in schizonts have a high degree of acetylated histone H3 at lysine 9 (H3K9ac) and that after the invasion, decreased levels of gene expression are accompanied by declines in the levels of H3K9ac and increased levels of histone H3 trimethylated at lysine 9 (H3K9me3), a marker of heterochromatin. However, genes transcribed in rings do not differ in levels of active H3K9ac/H3K9me3, regardless of whether they are active or silenced. Thus, the acetylation status of a gene is insufficient to establish transcription. Another report demonstrated that transcription factors PfTBP and PfTFIIE, part of the pre-initiation complex (PIC) of RNA pol II, remain bound to active genes during the erythrocytic cycle, regardless of the gene transcriptional status and acetylation levels at histones H3 and H4 [43].

Several nuclear markers have been characterized for *P. falciparum,* and further studies are necessary to characterize whether any of these are transcription sites markers as well. A more straightforward approach would be a functional characterization of *P. falciparum* RNA polymerases and their subunits, a fundamental undertaking that has still surprisingly not been achieved despite the relatively large number of studies on gene regulation and expression in *Plasmodium*, including some initial characterization work on RNA polymerases [44], [45].

In conclusion, the results presented here suggest that transcription in *P. falciparum* is organized in discrete foci, which resemble the transcription factories of higher eukaryotes. The transcription foci are dynamic structures that vary in number and distribution during the asexual cycle. Contrary to higher eukaryotes, the number of sites per *P. falciparum* nucleus does not appear to be related to the size of the nucleus (nuclear volume), and the intensity of transcription sites is higher in trophozoites than in rings. Given that evidence points to more active genes than sites observed in a given moment of the asexual cycle, it can be concluded that active genes would have to share sites while being transcribed. The nuclear subcompartment defined by transcription sites is different from some other compartments described previously for *P. falciparum*, and it exhibits low chromatin density.

This is the first study to address the spatial organization of global transcription in *P. falciparum* and to demonstrate that this nuclear subcompartment is dynamic and developmentally regulated. As the unique mechanisms of gene expression and regulation in malaria parasites raise the interesting possibility of targeting these pathways for drug discovery and ultimately malaria control, further studies are needed to address the issue of whether transcription sites are assembled upon the need for gene transcription or if they are pre-assembled structures, to which genes are targeted, stochastically or deterministically, to be transcribed.

## Supporting Information

Figure S1 **Nascent RNAs can be specifically labeled by BrUTP incorporation in** ***P. falciparum.*** Incubation of permeabilized *P. falciparum* cells with ATP, GTP, CTP and BrUTP allows for the detection of BrRNA by immunofluorescence (above, total transcription). The presence of the RNA polymerase II inhibitor α-amanitin blocks BrUTP incorporation into mRNA, and only nascent rRNA can be visualized in 1 or 2 spots per nucleus. When cells are incubated with BrUTP in the absence of ATP, GTP and CTP, no BrRNA can be visualized. Bars, 1 µm.
(TIF)
