# Supplementary figures and images for: Transcription Sites Are Developmentally Regulated during the Asexual Cycle of Plasmodium falciparum
Source: PLoS One. 2013 Feb 7;8(2):e55539. doi: 10.1371/journal.pone.0055539 (PMC3567098; doi:10.1371/journal.pone.0055539)

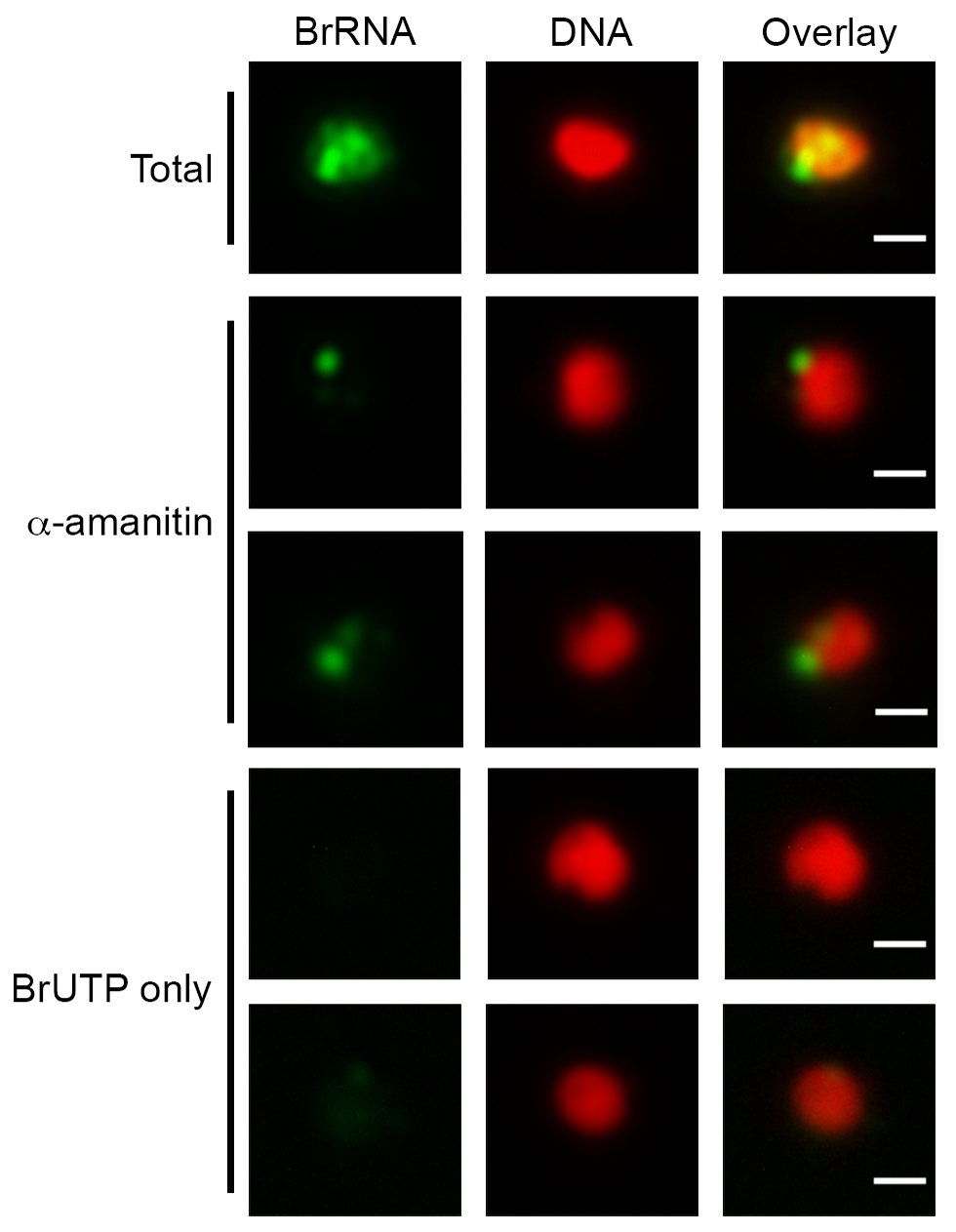

Supplement: Figure S1 — Nascent RNAs can be specifically labeled by BrUTP incorporation in P. falciparum. Incubation of permeabilized P. falciparum cells with ATP, GTP, CTP and BrUTP allows for the detection of BrRNA by immunofluorescence (above, total transcription). The presence of the RNA polymerase II inhibitor α-amanitin blocks BrUTP incorporation into mRNA, and only nascent rRNA can be visualized in 1 or 2 spots per nucleus. When cells are incubated with BrUTP in the absence of ATP, GTP and CTP, no BrRNA can be visualized. Bars, 1 µm. (TIF) [file pone.0055539.s001.tif]
